# Ignoring environmental change? On fishing quotas and collapsing coastlines in Bykovskiy, Northern Sakha (Yakutiya)

**DOI:** 10.1007/s13280-023-01874-9

**Published:** 2023-05-24

**Authors:** Olga Povoroznyuk, Peter Schweitzer

**Affiliations:** 1 Department of Social and Cultural Anthropology, University of Vienna, Schottengasse 10, 1010 Vienna, Austria; 2 Department of Social and Cultural Anthropology, University of Vienna, Universitaetsstrasse 7, 1010 Vienna, Austria

**Keywords:** Environmental change, Fishing, Infrastructure, Permafrost, Republic of Sakha (Yakutiya), Socioeconomic change

## Abstract

The Indigenous village of Bykovskiy is located 40 km from Tiksi, the administrative center of Bulunskiy District (Ulus), in the northern part of the Republic of Sakha (Yakutiya), Russia. Founded as a Soviet fishing cooperative, it became home to Indigenous Sakha, Evenkis, Evens, as well as to Russian settlers and political prisoners from the Baltic states. Post-Soviet transformations, coupled with escalating environmental change processes, has been altering the local economy and subsistence activities since the 1990s. Although our interlocutors directly observed and experienced such changes, they seemed to ignore the visible problem of severe coastal erosion that was destroying a local cemetery. This article is based on ethnographic fieldwork conducted in the study region in 2019, and combines approaches from the anthropology of climate change with reception and communication studies. It examines “ignorance” as a strategy of adaptation to multiple stressors under historically reproduced colonial structures of governance.

## Introduction

When we approached the village of Bykovskiy on the Russian Arctic coast in a small motorboat in August 2019, we were greeted by sights of eroding coastlines, most spectacularly by a graveyard being washed away into the sea. Over the following days, we had the chance to explore these visual witnesses of environmental change in more detail from the land, accompanied by local residents who made sure that we could see and document these signs of permafrost thaw. As social scientists, our attention, however, was focused less on the physical changes in the environment and more on how people perceived and talked about them. To our surprise, the people of Bykovskiy we talked to paid very little attention to collapsing coastlines and other effects of permafrost thaw and/or environmental change in their narratives. Instead, their focus was on the socio-economic challenges of post-Soviet life, on fishing regulations, and other forms of interactions with the state.

The aim of this article is to understand this seeming contradiction: while in many parts of the Arctic, as well as in other regions of the world, the discourse about environmental change—and even about change more generally—is dominated by the trope of climate change, the residents of Bykovskiy seemed to devote little attention to these rather dramatic transformations of the natural environment. Notwithstanding the provocative first line of the title of this article, we are not implying that local residents are ignorant or do not understand what is going on. On the contrary, they specifically pointed out to us sites with visible impacts of permafrost thaw. We were obviously not the first and only ones “hunting” for them. Over the years, members of international groups working at the nearby German research station in the Lena Delta region had stopped by in Bykovskiy and had made clear that these scientists were interested in climate change. We were no exception to the rule, despite being social scientists, as we visited Bykovskiy in the context of the H2020 project *Nunataryuk*, which is devoted to “permafrost thaw and the changing Arctic coast”.

When researching the local dimensions of climate change, anthropologists and other social scientists have often focused on the impacts of environmental change triggered by anthropogenic climate change. So-called reception studies have been relatively rare and typically constituted a late addition to the range of research focus areas geared toward climate change (de Wit and Haines 2022). Peter Rudiak-Gould (2011, 2014) has been among the pioneers of reception studies in anthropology, reminding social scientists that it is not enough to focus on how individuals and communities “observe” climate change but that we need to include the local reception of the science discourse of climate change into our studies as well (Rudiak-Gould 2011). Russia is of particular interest here, as there is a long history of official neglect or downplaying of anthropogenic climate change impacts (see, e.g., Wilson Rowe 2009). When Susan Crate started to conduct climate change research in the Republic of Sakha in the 2000s, reception of the (western) scientific discourse about these phenomena was largely and notably absent in the communities she worked (Crate 2008). While the topic of drastic environmental change has since received increased research attention in the Russian Arctic as well, anthropogenic climate change is typically not a prominent topic of public discourses in rural Siberia. In this article, we approach environmental change on the local level by combining elements of observation and reception studies. We explore how observations about environmental change are being communicated (or not), and which role climate change plays in the concert of issues facing a particular community in a historical perspective.

This article draws on a larger ethnographic field study that included community meetings, interviews with local residents and experts, and work with regional and district archival records in the Republic of Sakha (Yakutiya) in July and August 2019. As a result, a body of qualitative data, including observations, twenty-two interviews and three focus groups, as well as regional and district socio-economic development reports and plans were gathered in Yakutsk, Tiksi and Bykovskiy. Expert interviews conducted at the Melnikov Permafrost Institute, at the Ministry of Agriculture, and at the Ministry of Arctic Development and Indigenous Affairs of the Republic of Sakha (Yakutiya), and at the regional office of the Russian Association of Indigenous Peoples of the North (RAIPON) in Yakutsk, as well as conversations with experts from local organizations (the administrations, the hospital, the sea and the river ports in Tiksi and Bykovskiy) were important for gaining a deeper understanding of the environmental and social-economic impacts of permafrost degradation on the Arctic coastal communities, their local population, natural resource use and infrastructure. Ten biographical interviews with Indigenous^1^ residents and long-term settlers (both women and men in their 1930s, 1940s, 1950s and 1960s) from Bykovskiy, Tiksi and surrounding areas of Bulunskiy District were particularly valuable for our analysis of perceptions of permafrost thaw and environmental change. A focus group with Indigenous reindeer herders and fishermen from Bykovskiy and other villages of Bulunskiy District was conducted in Tiksi and addressed the losses caused by thawing permafrost for traditional land use and subsistence activities. Interviews and focus groups were transcribed and processed with the application of the method of content analysis. Statistical information on groups and dynamics of population, number of households and indicators of economic development (by main sectors) was additionally obtained from demographic and socio-economic reports prepared and provided to us by the administration of  Bulunskiy District and the local administrations in Tiksi and Bykovskiy. Unfortunately, follow-up fieldwork in the region has been impossible because of the spread of COVID-2019 and, since February 2022, because of Russia’s invasion of Ukraine. Therefore, the present article is based exclusively on the anthropological fielddata that were gathered in the region before the beginning of the pandemic and the war.

**Fig. 1:**
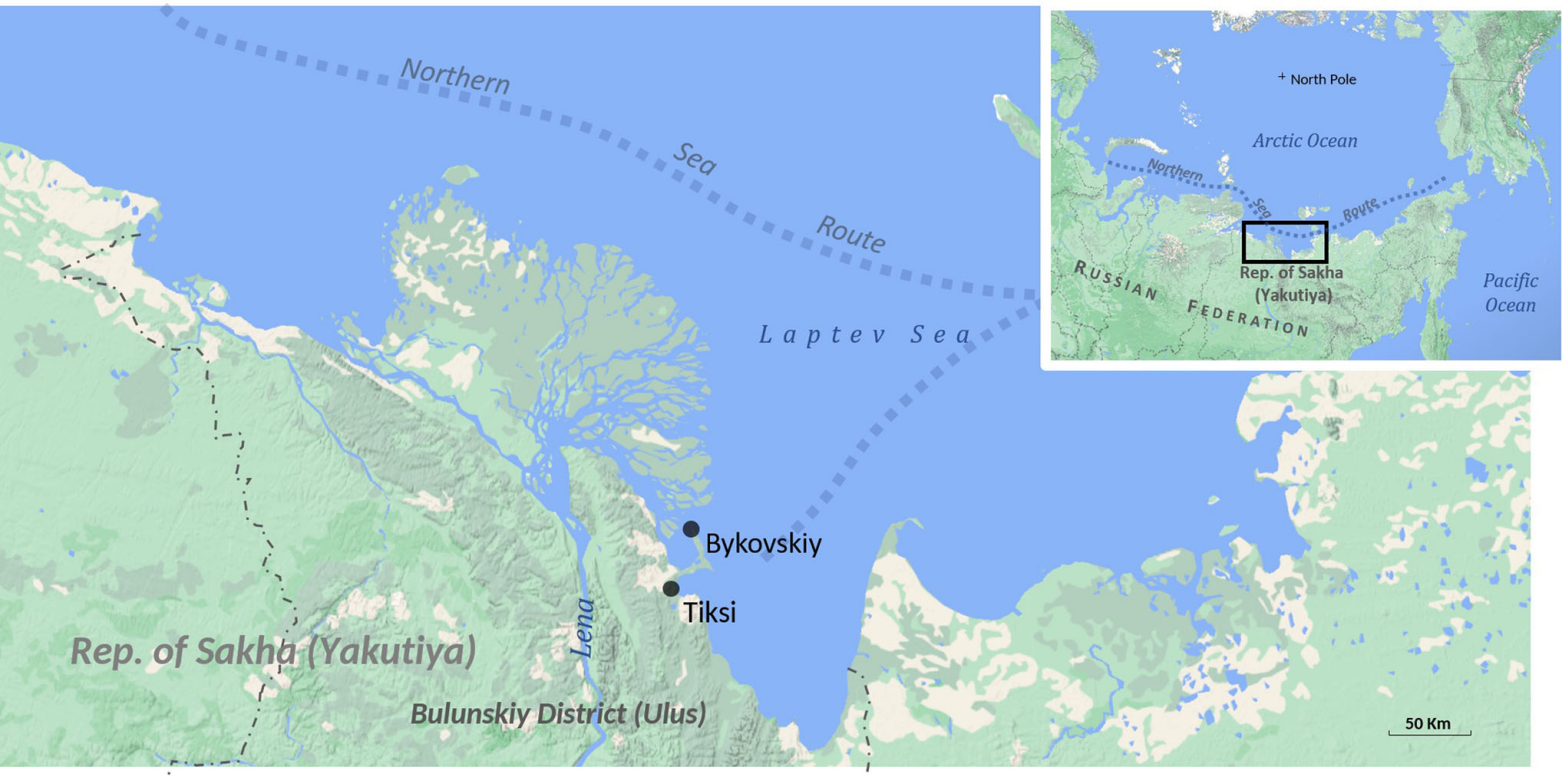
Location map with Bykovskiy and Tiksi in Bulunskiy District, Republic of Sakha (Yakutiya). Map by Alexis Sancho Reinoso

Figure 1 shows the location of Bykovskiy and Tiksi in relation to the rest of the Republic of Sakha (Yakutiya) and the Russian Federation as a whole, as well as the extent of the Northern Sea Route.

While many of our interviews and other research activities were conducted in Yakutsk and Tiksi, this article will focus primarily on Bykovskiy. The next section will introduce the collective farm “Arktika”, which has a dark past but provides the economic backbone of Bykovskiy untill the present day. This will be followed by a section on the post-Soviet realities of the village, which are characterized by collapsing coasts and diminishing fishing quotas. Another section will be devoted to portraying and analyzing how local residents live with and perceive changing environment. The conclusions will attempt to provide an explanation of the contradiction described above. We will analyze the uses and effects of modernization ideologies, environmental policies, and local knowledge along the Arctic coast to address the main question that prompted this article.

## Bykovskiy: Soviet collectivization and relocations

The rural community Bykovskiy is located on the Bykovskiy Peninsula in Bulunskiy District of the Republic of Sakha (Yakutiya), 40 km from Tiksi, the administrative center of the district. Its northern part stretches along the Laptev Sea coast, while its southern part faces Neelov Bay. It is the largest Indigenous fishing community in the area populated predominantly by Indigenous Evenkis, as well as by Evens, Sakha and Russians. This coastal territory has been for centuries occupied primarily by two closely related Tungusic-speaking groups—Evenkis and Evens who belong to the so-called numerically small peoples of the North. These two groups were originally leading nomadic lifestyles based on reindeer herding, fishing and partially on hunting. Semi-sedentary fishing groups of Sakha started migrating into the area since the late nineteenth century that is before the start of the large-scale Russian colonization of the Arctic cost, which began in the twentieth century.

The Soviet history of the region should be seen in the broader context of a national program of colonization, urbanization, and industrial development of the Arctic coast, on the one hand, and regional development and agricultural reforms unfolding in the Soviet Republic of Yakutiya, on the other. The Soviet program of “development of the North” (Slavin 1982) became an extreme example of modernization, including technological and social engineering, belief in progress and application of high technologies, as demonstrated by a series of large-scale infrastructure projects or “projects of the century” (*stroyki veka*) (Josephson 1995). One particular feature of Soviet modernization policies and ideologies was the almost complete neglect of environmental costs and the justification of destructive practices of “mastering” of fragile landscapes of the North as an inevitable price of progress (Josephson et al. 2013). The Northern Sea Route (NSR) became a mega-infrastructure project that completely transformed natural environments in the process of engineering new built and social environments in port towns such as Tiksi, and mixed rural settlements like Bykovskiy along the Arctic coast (Schweitzer et al. 2017).

Exploration and infrastructural development along the NSR played a remarkable role in the reconfiguration of transportation and supply in the Arctic. In Bulunskiy District, the earlier prevailing transportation system by reindeer shifted to full-scale provisioning of coastal communities by sea in the 1920 and 1930s (Struchkov 2005, p. 136). At the same time, according to local residents, dog sleds continued to be used for fishing and transportation of small cargos up to the 1980s when the snowmobiles were widely introduced (Interview PK and LK 2019). With the development of river transport in the 1950s, the so-called “Northern supply”^2^ partially switched to the Lena River (Durova 2020). The development of polar aviation further connected many coastal communities to the regional capital of Yakutsk, and the bigger ones, such as Tiksi—directly to Moscow. Despite lacking year-round roads, in the late Soviet period communities along the NSR enjoyed a wide assortment of foods and goods that were said to be unavailable in many parts of the “Big Land”^3^ (Povoroznyuk 2019, pp. 5–6). Although Bykovskiy is not located along shipping lanes, its proximity to the sea and to the river port of Tiksi ensured stable community supply and connection with two capitals—the regional and the national one.

The foundation of Bykovskiy in 1940, along with a number of other rural communities was part of the Soviet history of collectivization and colonization of the North, that was accompanied by repressions and forced relocations. In Bulunskiy District, the collectivization process included large-scale confiscation of reindeer, fishing and hunting equipment from the families that were classified as rich (*kulak*) or suspected in participation in anti-Soviet protests. By 1931, collectivization covered 630 or 47% of households in the district. In total 908 people joined collective farms (*kolkhozes*) including 67 Russians, 439 Sakha, 304 Evenkis and Evens, 85 Yukaghirs and 13 Chukchis (Struchkov 2005, p. 131). Collectivization boosted the sedentarization of nomadic ethnic minorities—Evenkis and Evens—and had devastating impacts on their traditional ways of life and subsistence activities. Large-scale fishing and fish-processing was introduced in the kolkhozes and significantly changed traditional subsistence fishing. The establishment of six fish-processing workshops in the district, including one based in Bykovskiy, lead to a division of labor within the industry—into fishing and fish processing (Gorokhov 2002, p. 19). These two activities have been employing the overwhelming majority of the local residents in Bykovskiy ever since. Fishing became the leading and most profitable economic sector. Other activities, such as hunting for fur animals and waterfowl, the gathering of bird eggs, became auxiliary activities of individual households, while the introduction of fur farms did not succeed.

The dark past of the village and the whole area is also associated with forced relocations of groups of Sakha people from the central parts of the republic.^4^ Even more dramatic were large-scale Stalinist ethnic purges and deportations of people from the Baltic states, the Volga region and the North Caucasus, to the Arctic coast during the same turbulent period. In 1942, 6000 out of total 74 400 deported people, were exiled to Yakutiya (Gorokhov 2002, pp. 19–20). It was primarily the residents relocated to Bykovskiy from other regions and Soviet republics who contributed to the development of fish-processing, while collectivized local Indigenous residents were involved primarily in large-scale fishing. The cultural and social division between local and relocated residents, among whom deported Finns and Lithuanians were especially visible, remained even though it was often masked behind the images of a peaceful multiethnic co-existence in Bykovskiy, which was constructed for public purposes vis-à-vis visitors (Stammler et al. 2017, pp. 8–9). Due to extremely challenging living and working conditions under constant surveillance, practically all deported residents died in Bykovskiy and were buried on a separate lot of land facing the Laptev Sea. In 1991, a monument to the victims of the Stalinist deportations was finally erected in the middle of the village by a Lithuanian NGO.

**Fig. 2:**
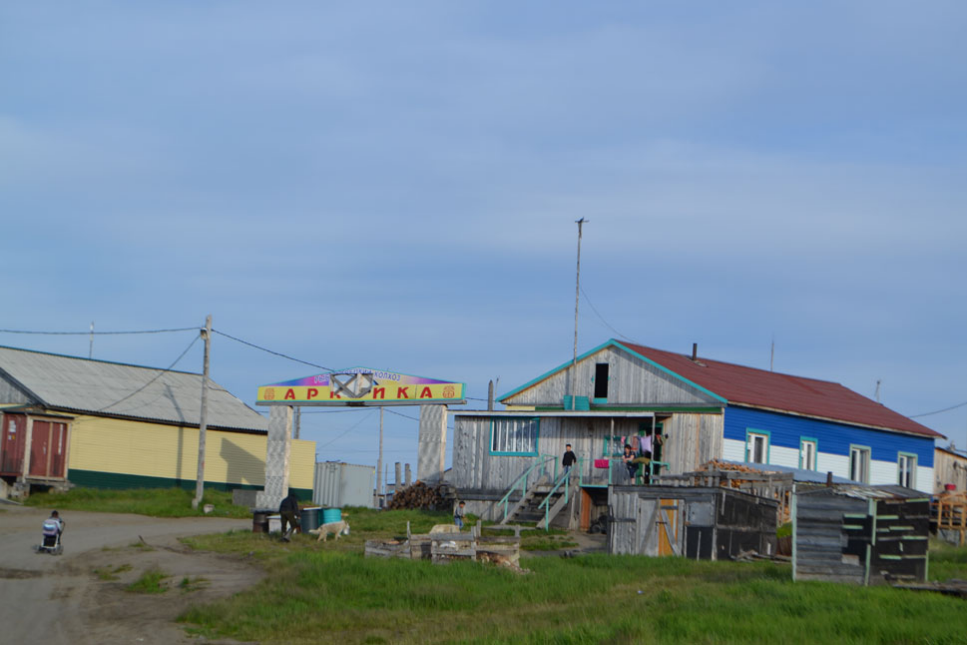
The sign "Arktika" in the center of Bykovskiy refers to the community-shaping fishing cooperative still in existence. Photo by Olga Povoroznyuk

Thus, the community of Bykovskiy survived the dramatic collectivization and relocations during the 1940, the closure of the fish-processing workshop in the 1980s, and the population outflow and drastic post-Soviet socio-economic transformations of the 1990s. While the fishing kolkhoz changed its name to “Arktika” in the 1950s, it retained its collective form of property and remained the main employer providing local residents with stable incomes in the uncertain period of economic transition. Our interlocutors, especially older generation residents of Bykovskiy, had nostalgic visions of the Soviet past and tended to stress the uniqueness of the fishing enterprise that has shaped community identity and provided for its economic well-being for many years (Povoroznyuk 2019, pp. 9–10) (Fig. 2).

## Post-Soviet realities: Living and fishing in remoteness

Post-Soviet socio-economic and political transformations have been affecting trajectories of development of Russia’s Arctic regions and lives of their permanent residents in multiple ways. Dramatic institutional changes resulted in new forms of organization, property and land use in agriculture and transformation of land use and subsistence activities of Indigenous peoples (Beach 2009). Socio-economic decline and withdrawal of state support, degradation of urban and transportation infrastructures, shrinking social services and rising communal and consumer prices have led to massive population outflow from the North (Heleniak 1999). Partial cancellation of local flights and of the state sponsored system of provisioning (*severnyy zavoz*) drastically reconfigured the connectivity between the Arctic coast and the country’s mainland. These changes have also affected the Republic of Sakha (Yakutiya) and its remote coastal communities which were suddenly left to survive on their own on the margins of the state. While Tiksi and neighboring communities of the Lena River delta were proud of their direct connectivity with Moscow during Soviet times, Yakutsk has been growing into the dominant economic and political center for this coastal area since the 1990s (Stammler et al. 2017). Currently, most of the goods are delivered to Tiksi by the Lena River during the river navigation period, or by ice road in the winter, while supply by the sea is limited to fuel and a few bulk items.

In the post-Soviet period, the economy of Bulunskiy District has been shrinking because of a decrease in jobs and challenges faced by private businesses. In the economic structure of the district, resource extraction (gold, diamonds, coal, construction materials and, recently, mammoth tusks) prevails. The agricultural sector is represented primarily by fishing, as well as by hunting, reindeer herding, horse breeding, and fur farming. Municipal enterprises (MUP), agricultural farming enterprises (KFKh), and other private collective and individual forms of enterprises and Indigenous *obshchinas,* came to replace kolkhozes and sovkhozes. The ‘kolkhoz Arktika’, which preserved its old name, is presently based on the rather unique legal form of a fishing cooperative (*artel’*) (Investitsionnyy Pasport 2017, pp. 5–7, 16).

The population dynamics of Bulunskiy District reflect the cycles of socio-economic development of the area. In 1989, the total population of the district constituted 17 630 residents, while by 2016 it dropped to 8366 people (Gukov 2013, p. 23, 26; Investitsionnyy Pasport 2017, p. 4). According to the head of the district administration, in 2018–2019 outmigration was, for the first time after a long period of steady population decline, compensated by in-migration (Interview IK 2019a). In 2017, Bykovskiy had 526 residents, including 187 men, 193 women and 146 children, living in 126 households (Naselenie 2017, p. 1). with the majority of them being Evenkis, Sakha, and Evens. With the beginning of the post-Soviet transformations in the 1990s, the village saw many local families leaving. Yet, according to long-term residents, the population loss here never reached the same scale as in Tiksi or other parts of the republic. Throughout this difficult period, fishing secured a rather stable source of food and income for people remaining in the village.

The existing cultural diversity of the region and the village is a subject of pride of local residents who associate it with “northern hospitality” to newcomers, both voluntary and forced ones. Despite a long history of interethnic marriages and cooperation and Russification of the area, interlocutors still draw the lines between locals and Sakha, as well as between locals and “the Balts”. Sakha, the titular ethnic group in the republic, are portrayed by other groups as colonizers and warriors that have been prospering off the lands and resources taken away from “genuinely” Indigenous peoples. Memory of “the Balts” as a past community remains, today embodied in a few descendants left, but, first and foremost, in the cemeteries of exiled Lithuanians, Estonians and Finns in Bykovskiy and on the neighboring Tit-Ary Island in the Lena Delta. During *perestroika*, relatives of the buried exiles came to visit these places; cases of exhumation and reburials are known; and a monument to the victims of the Stalinist repressions was erected in the center of Bykovskiy by a Lithuanian NGO. However, in the village itself, there is no one left to take care of the graves.

While the socio-economic crisis following the dissolution of the Soviet Union affected Bykovskiy, according to local residents, the overall quality of life remained unchanged in comparison to neighboring communities. This is how a local woman, an Indigenous activist and amateur writer, remembers the transition period in the village:

We have somehow managed to endure the rationing systems easier. When others were standing in lines to obtain their food stamps, the *kolkhoz* allocated to us sacks full of fish, flour or sugar ... we did not have those hardships [that others experienced]. The *kolkhoz* was always there (Interview ND 2019b).

Thus, one of the secrets of rather successful community adaptation to radical socio-economic transformations in the 1990s is associated with the stability of the local economy organized in the framework of the “kolkhoz Arktika”. The “kolkhoz” is, in fact, a legally registered fishing cooperative with a board of administration that makes decisions collectively. According to our informants, few key employees of “Arktika” have permanent full-time jobs, while most of the personnel is employed on temporary contracts that do not include social benefits typical for Northern regions. Still, opportunities to sell fish informally in addition to the basic income guaranteed by the enterprise, allows employees a decent standard of living (Interview ND 2019b).

According to the oral communication of the local administration in Bykovskiy, in 2019, the enterprise provided employment to 182 local women and men in fishing and fish-processing. In the summer season (in July and August), five to six fishing brigades catch fish in the sea waters as far as 40 to 220 km away from the village in the same fisheries that had been allocated to the cooperative in Soviet times. With the disintegration of the local fish-processing workshop, most of this business was lost to outside enterprises. Presently, most of the catch is sent to a large processing enterprise in Yakutsk that produces salted and smoked fish (Interview AYa 2019).

The “kolkhoz” inherited from its predecessor a number of facilities including a garage, a barn and a natural freezer built in an ice sheet by Soviet exiles. Due to changes in the permafrost, the freezer that for a long time has been successfully used for preserving fish, is losing this capacity. In 2018 and 2019, the amount of winter catch was so large that around 40 tons of fish could not be sold on the market. This situation led to a public discussion whether to purchase a refrigerator to store the remaining fish (Interview Aya 2019).

Being an important biological resource and, to a certain degree a commercial item, fish has remained a vitally important food product for local residents in Bykovskiy. Fishing is thus a subsistence activity that defines the traditional way of life of the community. At the same time, with the transition to a market economy, the Russian government introduced a number of new regulations affecting subsistence activities. For example, since 2004, fishing has been restricted by quotas and seasons, and the use of predefined types of gear has been prescribed (Ksenofontov et al. 2017, p. 291). Thus, the issue of quotas has become a new and challenging post-Soviet reality for local residents in Bykovskiy. The federal fishing agency *Rosrybolovstvo* that is responsible for the allocation of quotas, holds new auctions only in cases when large enterprises are liquidated. Thus, individual fishermen in Bykovskiy buy quotas from the “kolkhoz” with the obligation to catch and supply the amount of fish in question for a fixed low price to “Arktika”.

In practice, however, many people try to go beyond the limits set by quotas in order to sell the fish directly to consumers at a better price. This informal fishing is the main source of income for local households and is practiced by residents of all genders and generations. The Russian legislation foresees substantial fines and the confiscation of property for informal fishing and sale of its products, which are classified as illegal entrepreneurship and the illegal harvest of biological resources. Interlocutors recalled extreme cases of detainment of local fishermen, who were suspected of illegal fishing and brought from their fishing grounds dozens and hundreds of kilometers away directly to the administrative center of Tiksi for clarification of their cases by *Rybinspektsiya,* the main government agency controlling fishing. Having proven their right to fish, such unlucky fishermen would have to organize and pay for their way back to their fishing grounds in order to save the abandoned fishing equipment from drowning or freezing, if the incident takes place in winter.

As mentioned earlier, the regional transportation system of the northeastern Sakha is closely tied to the Northern Sea Route and the Lena River, while ground transportation is possible only along ice roads. Bykovskiy has seasonal access: by boats in the summer and by ice road in the winter. The existing informal dirt road connecting the village to Tiksi is in a bad condition and is only sporadically used by ATVs. While living in such remoteness has obvious disadvantages, local residents seem to enjoy some of its important advantage. Limited access to the village of the fishing inspection allows for a wide range of informal fish catching and selling practices that would not be possible would the permanent year-round road to Tiksi be built. According to the head of the district administration, the still pending road construction project was discussed and rejected by local residents of Bykovskiy, who voted for redirecting the funds to other community needs.

## Collapsing coastlines: Perceiving and adapting to changing environments

Soviet environmental history is a vivid example of large-scale transformation and degradation of natural habitats in the process of accelerated industrialization and modernization of the Arctic (Schweitzer et al. 2017; Breyfogle 2018). Underlying Soviet practices of extreme technological and social engineering was the ideology of “high modernism” (Scott 1998), based on a strong belief in scientific knowledge and linear progress. For example, the discourses of “conquest of nature”, “struggle with the (hostile) environment” and similar were characteristic of development policies and programs and were even used in scientific research on permafrost (Chu 2018, p. 167).

More recent Russian official environmental discourses and policies reveal older historical patterns of human relations with nature and infrastructural development driven by resource extraction interests (Povoroznyuk 2022). Federal legislation has been prioritizing issues of national security, socio-economic development, and the continued “conquest” of the Arctic over preventing environmental pollution and degradation and climate change (Strategiya 2020). At the same time, some Arctic regions managed to introduce legislation recognizing the risks of anthropogenic environmental change. For example, the law “On the Preservation of Permafrost in the Republic of Sakha (Yakutiya)” addresses cryogenic risks to infrastructure and communities and highlights the need to monitor permafrost conditions (Ob okhrane 2018). However, the configuration of jurisdictions and distribution of financial resources among three different levels of government in Russia limit regional and local initiatives, thereby leaving mitigation decisions and actions to federal authorities (Durova 2020, p. 232).

Implications of global climate and environmental change in the Arctic have been for a long time the focus of natural scientists, while increasingly attracting attention from social scientists, including anthropologists (see, e.g., Crate and Nuttall 2009, 2016; Hastrup 2013). Despite the lack of a critical public discourse on climate change in Russia, the country and especially its Arctic regions, like Yakutiya, have a long tradition of researching its impacts, including the degradation of permafrost (Chu 2015). Recently, there have been a few attempts to introduce transdisciplinary collaborative methods in the Russian Arctic by involving local scientists and communities and by integrating scientific and local environmental knowledge (Crate and Fedorov 2013; Crate et al. 2017).

The Republic of Sakha (Yakutiya) has been exposed for a long time to anthropogenic environmental changes, including pollution, landscape destruction in the process of resource extraction and, most prominently, thawing permafrost. Transdisciplinary research conducted in consultation with local and Indigenous communities in several districts of the republic distinguishes several types of changes driven by rising seasonal temperatures. They include thawing permafrost; changing hydrological regimes of rivers and lakes; unpredictability of weather patterns; changing habitats and migrations routes of wild animals; shifting seasons, etc. (Ananicheva et al. 2021). Our interlocutors from Tiksi and Bykovskiy reported multiple instances of environmental change, including stronger winds and snow storms in the winter, earlier melting of sea ice in the spring, decreasing fish stock in the sea, shorter and unpredictable use periods of ice roads. These changes negatively impacted their health, subsistence practices, mobility options and the availability of fuel and food products.

Among other environmental changes, thawing permafrost has dramatic effects on local infrastructures, economies and ways of life in Sakha (Yakutiya). As a multidisciplinary study of *alaas* (thermocarst depressions with grassland areas) in central regions of the republic has shown, an unprecedented rate of permafrost thaw challenges the adaptive capacity of traditional animal husbandry in these keystone cultural areas (Crate et al. 2017). In Bulunskiy District, the degradation of permafrost destroys or impairs ice cellars, impacts the quality of fresh water and sea water, and jeopardizes the stability and functioning of transport and housing infrastructures. For example, the destruction of ice cellars endangers food security in remote rural communities such as Bykovskiy, as does the changing seasonality of ice roads, which are often the only means of connectivity and supply of vitally important fuel and food items (Schweitzer and Povoroznyuk 2022). While most of Soviet-era infrastructures in Tiksi remain stable, permafrost thaw has been contributing for decades to dramatic coastal erosion in Bykovskiy.

The coastline of the Bykovskiy Peninsula where the community is located, is characterized by the presence of ice-rich sediment. Between 1951 and 2006, the coast retreated at an average rate of 59 cm per year due to erosion caused primarily by thermokarst activity as well as by storms and other factors (Lantuit et al. 2011). The erosion has been affecting community infrastructures—public kolkhoz buildings and the communal ice cellar, as well as private houses of local residents. However, the most dramatic and visible outcome of the retreating coast in Bykovskiy is the destruction of the cemetery of the deportees of the Stalinist regime. Open graves and coffins with crosses sliding down along with the muddy ground into the ocean, were the first startling glimpses of the village as we were approaching it in a boat by sea. Our follow-up visits to the site located directly by the coast at the outskirts of the village confirmed the grim reality: the graveyard with dozens of burials, left without maintenance, became subject to destruction by the force of coastal erosion.

In our conversations, we repeatedly asked residents of Yakutsk, Tiksi and Bykovskiy about climate change, permafrost thaw and the issue of the cemetery in Bykovskiy, which—we assumed—should have preoccupied the minds of our interlocutors. However, to our great surprise, neither of these issues seemed to be of major concern. Our research has shown that most experts and residents in Sakha (Yakutiya) are aware of climate change impacts, have directly experienced them or have been living with them for a long time. However, their perceptions and framings of the observed climate and environmental changes appeared to vary between ignorance, indifference, and skepticism (Schweitzer and Povoroznyuk 2022). The predominant attitude of our interlocutors to the issue of the cemetery can be labeled as indifference. “We were playing football with the skulls [from the graves] as kids”, commented one middle-age local resident. Yet, our following in-depth conversation showed the complexity of the issue. Activists, journalists, as well as the local administration and residents of the village have been raising concerns about potential viral and organic contamination, as well as about spiritual and psychological problems connected with the destruction of the cemetery. Their voices are rarely heard at the federal level, where resources to manage such large-scale environmental disasters as coastal erosion are accumulated. “Without decisions made on the top level, we have no power to do anything”, stated the major of Bykovskiy. To our question about local reactions and possible actions regarding the cemetery a local Evenki resident and activist responded:

The cemetery will remain here as long as the [Bykovskiy] Peninsula exists. And what else one can do about it? Who will rebury the bodies? (Interview ND 2019b)

Some other local interlocutors didn’t have an opinion on the issue of the coastal erosion nor on the destruction of the cemetery. Even though their own houses were located close to the retreating coastline, they didn’t express immediate concerns about risks for their houses coming with the coastal erosion. What they worried about in our conversations about climate change, were rather declining fish stock, changing quality of sea and fresh water, less predictable fish migrations and more complicated ice conditions at sea than before. Coupled with growing competition for fishing quotas and tightening control over this key economic activity and access to biological resources altogether, these changes seemed to endanger culturally important subsistence activities and a way of life.

## Discussion and conclusion

As the previous sections have demonstrated, the residents of Bykovskiy are well aware of the environmental changes happening around them and do not “ignore” them because of the lack of knowledge. The changes most visible to us, as outsiders, such as coastal erosion, are locally perceived as everyday phenomena that people have been living with and adapting to for decades.

As mentioned above, Post-Soviet transformations since the 1990s have severely altered the economic base of northern communities, typically resulting in social, cultural and economic shocks and hardships. Bykovskiy seems to have weathered these transformations relatively well. Still, the village is a fishing community, where individual and communal well-being is highly dependent on the sea and its resources. While average residents may not be able to scale up local environmental changes to global processes, experiential indicators such as water quality or the availability and quality of fish, seem to be most relevant for them. In the context of shrinking fish stock and growing competition for marine resources, the distribution of fishing quotas, the market price for fish and other immediate socioeconomic concerns seem to trump long-term environmental issues, such as permafrost thaw and coastal erosion.

Indigenous residents, especially, those making a living by practicing “traditional” activities, such as fishing and reindeer herding, have been observing extreme weather events, shifts in seasonal patterns, and changes in the behavior of land animals and fish for a long time. Similar to other parts of the Arctic, this accumulated traditional ecological knowledge has been helpful for adapting to the dramatically changing environment. At the same time, on the discursive and political level, this knowledge has been devalued or, at best, rated as a secondary source of information in relation to a more “advanced” institutionalized expert knowledge. Moreover, the Soviet modernization ideologies and discourses about human-environmental relations have impacted the production of knowledge about the changing environment.

Our observations correspond with what other researchers have noted in other parts of the Russian North (see, e.g., Crate and Fedorov 2013; Graybill 2013; Anisimov and Orttung 2019). The specificity of Russian (political and scientific) discourses about climate change has been discussed for some time (Crate 2008; Wilson Rowe 2009), without leading to definitive conclusions. Instead, a variety of factors seem to be at play at different levels and scales (including the idea that Russia might benefit from climate change). While standard analyses of the factors impacting the perception of climate change (see, e.g., Weber 2010) seem to highlight the importance of information, education and morality, our case—and similar ones—demonstrate that cultural knowledge systems and cognitive frameworks might be even more important. This is in line with research on cultural cognition (e.g., Kahan et al. 2011) and with recent U.S. case studies outside mainstream white culture (Naiman et al. 2023).

The tragedy of Russia’s invasion of Ukraine has highlighted one more point we want to make. While the 1990s and early 2000s, that is the first decade or two after the dissolution of the Soviet Union, were characterized by previously unknown processes of grassroots initiatives and local engagement in Indigenous (and non-Indigenous) communities of Siberia and the Russian Far East, the last 10–15 years—and especially the years since 2014, when Russia annexed Crimea—saw a progressive tightening of central control, which made local initiatives economically unfeasible and politically dangerous. In such a colonial and increasingly authoritarian context, “ignoring” could be seen as a strategy of ensuring individual and community security vis-a-vis multiple socio-economic and environmental stressors.

Finally, this case study is another reminder of the well-known caveat that the cultural assumptions and social biases of us, the researchers, should not cloud our understanding of other people’s thoughts, actions and agencies. In our ethnography of local perceptions of environmental change, we strive to acknowledge that the people of Bykovskiy are most knowledgeable about environmental and other processes in and around their community. Thus, this is another call for the integration of local observations and experiences into scientific models of climate change (compare Callaghan et al. 2020). In that sense, “ignorance” is not the absence of knowledge but a state of miscommunication between different types and scales of environmental knowledge. It should also be seen as a strategy of local adaptation to major socio-economic and environmental changes under colonial structures of governance.

## Footnotes

1 The Indigenous population of the region consists of Sakha (or Yakuts), Evenkis and Evens; their legal status, however, is differentiated by Russian legislation. The Sakha (Yakuts) belong to the numerically larger Indigenous groups of Siberia, their population exceeding the threshold of 50 000 people. Evenkis and Evens belong the category of “numerically small people of the North” and their indigeneity is recognized in the form of a number of social benefits, including—at least formally—the use of land and waters for subsistence activities.

2 A state regulated system of provisioning of local communities in the Soviet North and later in the Russian North that supplied food items, consumer goods and fuel.

3 The Russian notion *bol’shaya zemlya* is a vernacular expression used to signify the “mainland” of the country in opposition to its northern, remote communities.

4 People were relocated under the Soviet policy of amalgamation (*ukrupnenie*), which officially labeled remote rural settlements as being “without a perspective” or economically unprofitable and led to their closure throughout the 1940s and later.
